# Supplementary material for: Rapid turnover of life-cycle-related genes in the brown algae
Source: Genome Biol. 2019 Feb 14;20:35. doi: 10.1186/s13059-019-1630-6 (PMC6374913; doi:10.1186/s13059-019-1630-6)
Supplement: Supplementary file 3 — Protein alignments, corresponding Gblocks html file and CDS sequences for the lineage conserved gametophyte-biased genes. (ZIP 2109 kb) [file 13059_2019_1630_MOESM3_ESM.zip › Con_GA_alignments/Ec-01_011620.aa_ali.fasta-gb.htm]

Ec-01\_011620.aa.fasta.trans.aa\_ali.fasta


## Gblocks 0.91b Results

Processed file: **Ec-01\_011620.aa.fasta.trans.aa\_ali.fasta**  
Number of sequences: **2**  
Alignment assumed to be: **Protein**  
New number of positions: **180** (selected positions are underlined in blue)

```
                         10        20        30        40        50        60
                 =========+=========+=========+=========+=========+=========+
Ec-01_011620     MSATCGCVVVRRCIFIFPMIPEPSALGDLPLGALTEA-VSAESAAGPTCSSADEVDDDFS
MSTRG.13338.1    -----------------SMLPAISVLGNLPPDVLIEAERSSEGTRG--LPPQEGTADGCS
                                   ###################                   ####


                         70        80        90       100       110       120
                 =========+=========+=========+=========+=========+=========+
Ec-01_011620     ILKYEDPVLARPDIAFDMRMGHDSLPQYISLYASDERRPDEATQRLAGVGAAAPGSTRNT
MSTRG.13338.1    ILKYEDPVLVRPHASYDMALGHDSLPQYVSLYAANERGPVEGALHHSATSGVATRSIR-T
                 #########################################                  #


                        130       140       150       160       170       180
                 =========+=========+=========+=========+=========+=========+
Ec-01_011620     TRRPRGGGDDITAVLKAILPNKEYADAAGEWVQTVSGTVRSRGELEKVQNKVEARLATLQ
MSTRG.13338.1    KIRPSGGGADITAVIKAIIPNKEFSDGAGKWVQTISGSVRTRSELDKVQGEVDSQLAALQ
                 ############################################################


                        190       200       210       220       230       240
                 =========+=========+=========+=========+=========+=========+
Ec-01_011620     ARPGAVCAVRESVFSELYDEVIREITLQSPERGLLLLRLRDETRMNLDTYAEIYR-----
MSTRG.13338.1    ARQTPVCPVREQVLSELFDEVIREITLQCPERGLLLLRLRNETRMNLDTYAEIYRDSFAF
                 #######################################################     


                        250       260       270       280       290       300
                 =========+=========+=========+=========+=========+=========+
Ec-01_011620     --------------------CLMLPSCLFQMKKSVFTTELH----------------SII
MSTRG.13338.1    SRRKVEGAQQDLDGTRSLLIGLQRETTGLQTRVRSLEADLASINRSNAYTRSATDHEHIE
                                                                             


                 
                 ========
Ec-01_011620     FDRRTVIN
MSTRG.13338.1    TLRLTKEE
```

```
Parameters used
Minimum Number Of Sequences For A Conserved Position: 2
Minimum Number Of Sequences For A Flanking Position: 2
Maximum Number Of Contiguous Nonconserved Positions: 8
Minimum Length Of A Block: 10
Allowed Gap Positions: None
Use Similarity Matrices: Yes
```

```
Flank positions of the 3 selected block(s)
Flanks: [19  37]  [57  101]  [120  235]  

New number of positions in Ec-01_011620.aa.fasta.trans.aa_ali.fasta-gb:  180  (58% of the original 308 positions)
```
